# Mycotic Aortic Aneurysm: A Rare Etiology of Septic Shock

**DOI:** 10.7759/cureus.24376

**Published:** 2022-04-22

**Authors:** Kaitlin M Bowers, Vishnu Mudrakola, Christopher M Lloyd

**Affiliations:** 1 Emergency Medicine, Campbell University School of Osteopathic Medicine, Lillington, USA; 2 Emergency Medicine, Licking Memorial, Newark, USA; 3 Emergency Medicine, OhioHealth Doctors Hospital, Columbus, USA

**Keywords:** aortic aneurysm surgery, complicated urinary tract infection, e. coli, mycotic aortic aneurysm, pathophysiology of septic shock

## Abstract

Mycotic aneurysm of the aorta is a rare disease with a high mortality rate due to its likelihood of aneurysmal rupture. This syndrome is predominantly seen in patients over age 65 with the most common presenting symptoms being fever and back pain. Our case illustrates a mycotic aneurysm of the aorta presenting in an elderly female with vague abdominal pain, flank pain, and generalized weakness. We review the investigative approach, diagnostic modalities, and treatment options in patient management. This case emphasizes the need for a high index of suspicion of mycotic aneurysms of the aorta in critically ill elderly patients as early antibiotic therapy can be crucial for source control.

## Introduction

Mycotic aneurysm of the aorta (MAA) is an aortic aneurysm caused by infection. This terminology was first used by Sir William Osler to describe this subset of aneurysms as a fungal-like growth in his Gulstonian lectures in 1885 [1]. While the term mycotic suggests a fungal etiology, Osler connected mycotic aneurysms to bacterial origin through his use of gram stain in his studies of endocarditis. Despite the discovery of both bacterial and fungal causes, the term mycotic has remained. Although there is a push to standardize the term infected aneurysm of the aorta, for the purpose of this case report we will continue to refer to this disease process as MAA [2].

MAA is an extremely rare condition with a varying global prevalence. In two studies reviewing autopsies performed at Boston City Hospital and Mayo Clinic, MAA was reported in only 1%-1.5% of all cases of aortic aneurysms [3,4]. These studies also revealed a high rate of aneurysmal rupture highlighting the importance of a high index of suspicion and early diagnosis. A majority of the initial reported MAA cases were associated with endocarditis [5]. The introduction of organism-specific antibiotics has decreased the prevalence of MAA-associated endocarditis. However, new risk factors for the development of MAA, primarily the increased frequency of intra-vascular procedures and intravenous drug abuse, have led to a resurgence of cases [6].

MAA can occur at any point along the thoracoabdominal aorta [7]. There is a three times higher incidence in men and MAA also tends to primarily affect patients over the age of 65. Other strong associations include tobacco abuse and diabetes mellitus [8].

The classic triad of fever, abdominal pain, and pulsatile mass is a rare finding in patients presenting with MAA. Rather, the majority of the symptoms are nonspecific with back pain and fever being the most common presenting features [9]. Before the antibiotic era, Gram-positive cocci implicated in endocarditis were the most common causes of MAA. Gram-negative organisms now account for up to 40% of cases with the predominant pathogens being *Staphylococcus aureus* and non-typhoidal *Salmonella* species [9,10]. *Escherichia coli* (*E. coli*) is a less common cause but also has been associated with mycotic aneurysms [9-11]. True fungal infections causing MAA are extremely rare. Only seven cases of fungal causes of vascular aneurysms, in general, were reported from 1966 to 1999 [12]. We describe a case of an 82-year-old immunocompetent female who presented with vague symptoms and was found to have an MAA caused by *E. coli* bacteremia.

## Case presentation

An 82-year-old female presented to the emergency department (ED) via ambulance complaining of malaise, myalgias, and abdominal pain. Paramedics reported the patient's family called them for “altered mental status”; however, the patient had been answering questions appropriately. Upon arrival, she was alert but disoriented to time. The patient reported three hours of generalized body aches, weakness, and abdominal pain. She described her pain as constant, sharp, and localized to her right abdomen and flank. She admitted to occasional episodes of non-bloody, non-bilious emesis throughout the past week, but she had experienced none on the day of evaluation. She denied fevers, chills, cough, chest pain, shortness of breath, dysuria, or diarrhea. She did report a history of chronic back pain but stated this was no worse than normal.

The patient had a past medical history of type 2 diabetes, stroke, chronic obstructive pulmonary disease, congestive heart failure, hypertension, hyperlipidemia, lumbosacral disc disease, and remote deep venous thrombosis. Her surgical history included prior back surgery and hysterectomy. She had a thirty pack per year smoking history, and she denied alcohol or illicit drug use. She had no pertinent family history.

Presenting vital signs were blood pressure of 74/45 mmHg, heart rate of 86 bpm, respiratory rate of 18, oxygen saturation of 94% on room air, and an oral temperature of 97.9°F. The patient appeared cachectic, frail, and disheveled. She had coarse breath sounds bilaterally and no signs of respiratory distress. Her abdomen was soft with right-sided tenderness to palpation, but no rebound or guarding. She had 2+ pulses that were equal in all four extremities. She followed all commands and answered all questions appropriately but continued to be disoriented to time. There was no family present to confirm her baseline mental status.

Given the patient’s hypotension and reported history of altered mental status, a septic workup was initiated. She received a 30 mL/kg bolus of normal saline. Laboratory analysis revealed a leukocytosis of 41.11 and a lactate of 2.8. The patient was started on broad-spectrum antibiotics, Vancomycin, and Cefepime, for septic shock. The complete metabolic panel revealed hyponatremia (sodium 129), hypokalemia (potassium 2.1), hyperglycemia (glucose 302), and acute kidney injury (creatine 2.61). However, the corrected sodium was 132. The patient's potassium was replaced with 80meq parenterally through a peripheral IV.

**Figure 1:**
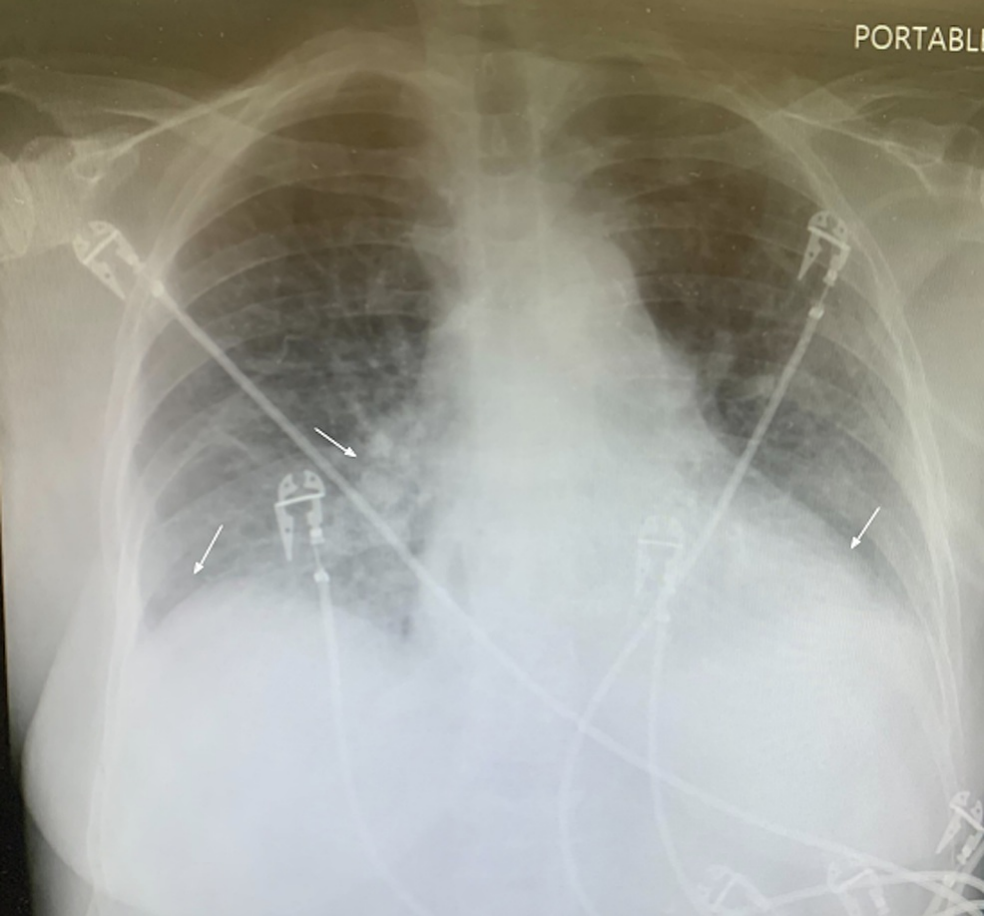
Portable chest x-ray demonstrating right lower lobe atelectasis, pulmonary vascular congestion, and cardiomegaly (arrows).

The patient’s urinalysis, obtained via catheterization, showed trace leukocyte esterase with 95 white blood cells (WBCs) and many bacteria, consistent with a urinary tract infection. One-view chest x-ray (Figure 1) was reported by radiology as mild cardiomegaly with mild pulmonary vascular congestion, mild right basal atelectasis, and questionable small bibasal effusions. CT imaging of the brain demonstrated no acute intracranial abnormalities.

**Figure 2:**
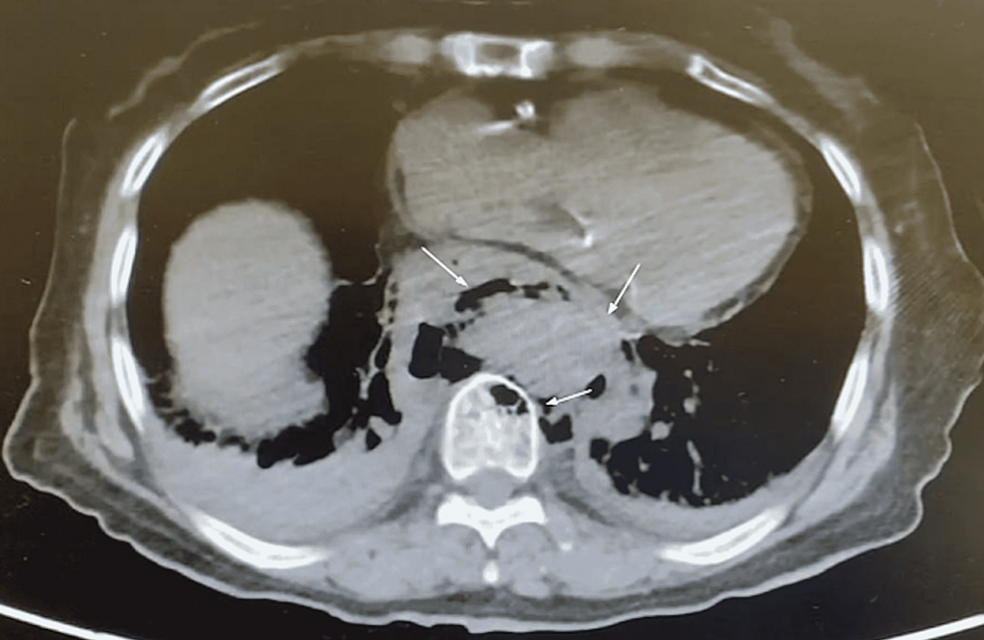
CT abdomen and pelvis without contrast. Air-fluid levels are apparent in the mediastinum as well as the vertebral body. Aortic involvement is also highlighted (arrows).

CT abdomen and pelvis without contrast (Figure 2) were ordered due to the patient’s complaint of abdominal/flank pain in the setting of septic shock. Imaging showed a large complex collection of gas and fluid situated in the posterior and inferior mediastinum surrounding the distal esophagus and aorta with extension into the marrow cavities of the adjacent vertebral bodies. No acute intra-abdominal pathology was noted. These findings were concerning for mediastinal abscess versus esophageal rupture.

**Figure 3:**
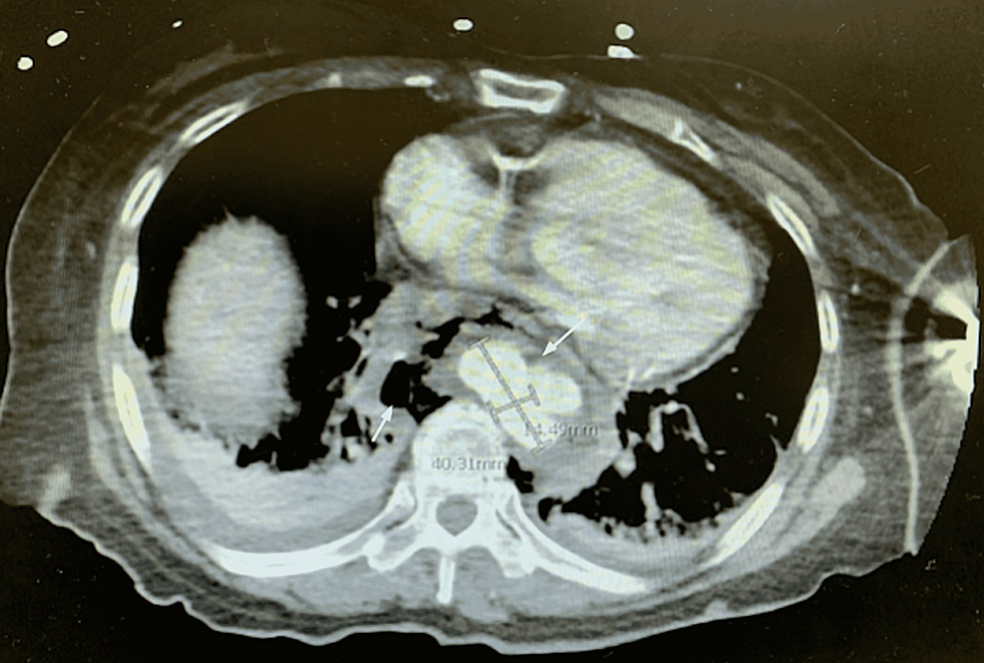
CT chest with contrast demonstrating a pseudoaneurysm of the aorta (measurements) and air-fluid levels in surrounding tissues (arrows).

Upon reassessment, the patient denied any chest pain, shortness of breath, or difficulty swallowing. She also denied any recent procedures or instrumentation. The patient was taken urgently back to CT for a scan of her chest with contrast to better evaluate the extent of her abnormal findings. CT chest with contrast (Figure 3) showed a large pseudoaneurysm of the right descending aorta with an extraluminal collection of contrast that measured 3.2 by 4.0 by 1.5 cm. The surrounding tissues were reported to show an admixture of fluid and gas, suggesting a retroperitoneal abscess, or potentially esophageal rupture. Due to concern for an abscess with vertebral involvement Flagyl was added for additional anaerobic coverage.

The patient remained hypotensive despite large volume of fluid resuscitation and therefore a femoral central venous catheter (CVC) was placed. The patient was started on norepinephrine and transferred to a tertiary care center with cardiothoracic capabilities.

At the tertiary center, the patient had a multidisciplinary approach with evaluation and recommendations from cardiothoracic surgery, vascular surgery, infectious disease, and critical care. An esophagram was performed and was negative for esophageal injury. The patient was ultimately diagnosed with mediastinitis, pneumomediastinum, septic shock, and vertebral osteomyelitis secondary to a mycotic aneurysm of the descending aorta. Vascular and cardiothoracic surgery did not feel there were any reasonable surgical or endovascular options given the extent of her infection and her advanced age. Surgical debridement or drainage of her mediastinum would likely cause a rupture of her pseudoaneurysm. There was also concern that given her infectious extension into the spine any attempts at debridement would likely be unsuccessful. Repair of the aneurysm itself would require an extensive thoracoabdominal approach as any attempts at endovascular stenting would immediately be subject to infection. Given the extensive surgery that would be required surgery recommended palliative antibiotics without surgical intervention. 

Blood cultures were positive for *E. coli* bacteremia and the patient’s antibiotics were narrowed to ceftriaxone. She continued to require vasopressor support with norepinephrine and subsequently went into hypoxic respiratory failure requiring a high-flow nasal cannula. Hospice was consulted and the patient’s code status was changed to comfort care. Unfortunately, the patient succumbed to her disease process two days after her initial ED presentation.

## Discussion

The diagnosis of MAA requires a high index of suspicion and early recognition of risk factors due to its nonspecific presentation [13]. There are no specific laboratory findings that are highly suggestive of the diagnosis, however, leukocytosis and other inflammatory markers such as erythrocyte sedimentation rate (ESR) and C-reactive protein (CRP) have been noted to be abnormal. It is also important to obtain blood cultures as they are positive in 50%-90% of cases [8]. Imaging is key to establishing the diagnosis. The American Heart Association recommends computed tomography angiography (CTA) of the aorta as the initial imaging modality of choice. CTA is a rapid test that allows for the identification of the aneurysm, location, and risk of impending rupture [13]. 

MAA in the setting of *E. coli* bacteremia is very rare. As discussed earlier there is a shift in causative bacteria to gram-negative organisms due to better recognition and treatment of endocarditis. Due to the overall rarity of MAA, a clear prevalence of *E. coli* bacteremia as the cause has not been established. In a handful of case series, *E. coli* was a rare cause with non-typhoid salmonella being more common [9,11]. *E. coli* is an emerging cause of MAA, especially with increased recognition and treatment of organism-specific endocarditis. *E. coli* is also a very common cause of urologic infections and recently there has been a link drawn between severe urologic infections causing bacteremia and thus MAA [10]. Interestingly, our patient did have bacteriuria and pyuria suggesting a urologic process could have seeded the infection that ultimately resulted in MAA, but definitive urine cultures showed no growth.

With regard to the treatment of MAA, there is no standardized approach or general consensus due to the rarity of the condition and inherent difficulty in performing randomized controlled trials. Treatment generally is tailored to the patient and involves a multidisciplinary team approach to infectious disease, cardiothoracic surgery, and vascular surgery. Antibiotic therapy is initially broad until it can be focused on culture results. Surgical options include endovascular treatment and open resection with either extra-anatomic reconstruction or *in situ* reconstruction [13]. The optimal timing for surgical intervention is difficult to determine. Ideally, surgery would occur once the infection has been controlled. Some authors recommend serial CT scans to aid in deciding when to intervene surgically [14]. However, in those patients with a high risk of aneurysm rupture, emergent surgery is unavoidable [14]. Post-operative outcomes have been shown to be dependent on the anatomic location of the MAA and the severity of infection [9]. Antibiotic-only therapy is suitable for patients that are deemed not surgical candidates or those that choose to forgo surgery [8]. Long-term antibiotic coverage is recommended for at least six months; however, many surgeons choose to prescribe life-long oral antibiotics due to the high risk of recurrence [9,15].

MAA is a very rare condition and carries with it significant morbidity and mortality. Based on previous case series and retrospective reviews, overall mortality ranges between 15% and 75% [9,16-18].

## Conclusions

MAA is a very rare condition that carries significant morbidity and mortality. Due to many factors, including better treatment of endocarditis and increased complications associated with urologic infections and procedures, gram-negative organisms such as *Salmonella *and *E. coli* have become more common causes of MAA. As seen in our case, presenting symptoms are typically vague which can make early diagnosis challenging. Prompt antibiotic treatment can lessen the disease severity, due to source control, which in turn has been shown to improve postoperative outcomes. Thus, MAA is an important diagnosis to consider in the ED for critically ill elderly patients.
